# Sustained Regression of Parotid Mucosa-Associated Lymphoid Tissue (MALT) Lymphoma After Nutritional and Nutraceutical Intervention Without Chemoimmunotherapy: A Case Report

**DOI:** 10.7759/cureus.115791

**Published:** 2026-09-04

**Authors:** Mark Simon, Amilcar Exume, Tomer Egozy

**Affiliations:** 1 Nutrition, Nutritional Oncology Research Institute, Westlake Village, USA; 2 General Surgery, Kaiser Permanente Medical Center, San Diego, USA; 3 Family Medicine, Hashmura Clinic, Zikhron Ya'akov, ISR

**Keywords:** alkalinization, cysteine restriction, ferroptosis, methionine restriction, mucosa-associated lymphoid tissue (malt) lymphoma, nutritional oncology, parotid lymphoma, reactive oxygen species (ros) in cancer, sjogren’ s syndrome, sodium selenite

## Abstract

Extranodal marginal zone lymphoma of mucosa-associated lymphoid tissue (MALT) in the setting of Sjögren's syndrome (SS) is a well-recognized complication of chronic autoimmune salivary gland inflammation. Standard management options include radiotherapy, immunotherapy, and combination chemoimmunotherapy, with active surveillance reserved for stable, low-burden disease. The potential role of structured nutritional and nutraceutical intervention in this setting has not been previously described. We report the case of a woman who presented at age 52 with SS-associated bilateral parotid MALT lymphoma that progressed substantially over 28 months of active observation, with palpable bilateral parotid enlargement reaching 10 x 5 cm on the left and 6 x 6 cm on the right. Proposed chemoimmunotherapy was deferred by the patient, and she independently initiated a structured multicomponent protocol comprising a strict vegan diet restricted in methionine and cysteine, urinary alkalization, and orally administered nutraceuticals including therapeutic-dose slow-releasing sodium selenite (32 mg/day), B6 pyridoxal-5'-phosphate (P5P), iron, zinc, copper, and manganese, with adjunctive rectal ozone therapy started at approximately Month 3. Serial clinical examinations and ultrasonography documented progressive bilateral parotid regression from approximately Month 7. At Month 20, repeat ultrasonography confirmed substantial radiographic reduction: the right parotid lesion decreased to 1.5 × 0.8 cm and the left to 2.7 × 0.8 cm, with both parotid glands returning to normal overall size with residual hypoechoic lesions and no cervical lymphadenopathy. At approximately Month 19, the hematologist documented no significant lymphadenopathy or splenomegaly on serial physical examination. Prednisone dosing, prescribed for concurrent autoimmune hemolytic anemia (AIHA), fluctuated independently of parotid gland changes throughout, making a corticosteroid-mediated response unlikely. This case documents a rapid and durable regression of progressive bilateral parotid MALT lymphoma after a dietary and nutraceutical protocol targeting tumor redox biology, without chemoimmunotherapy. Although spontaneous regression cannot be excluded, the response timing and proposed mechanism of glutathione and thioredoxin antioxidant pathway disruption merit further clinical study.

## Introduction

Extranodal marginal zone lymphoma of mucosa-associated lymphoid tissue (MALT) is an indolent B-cell non-Hodgkin lymphoma (NHL) that arises at epithelial sites subject to chronic antigenic stimulation (persistent local immune activation), most commonly in the context of persistent inflammatory or autoimmune conditions. Its association with Sjogren's syndrome (SS) is well established: SS drives sustained B-cell activation and an exaggerated ectopic germinal center reaction, hypothesized to promote clonal B-cell transformation [1], and patients with SS carry a markedly elevated risk of developing B-cell lymphoproliferative disorders, with a standardized incidence ratio for NHL estimated at about 18 times the general population. Marginal zone lymphoma of the major salivary glands, particularly the parotid gland, represents the most frequent subtype [1].

Management of parotid MALT lymphoma is risk-stratified and often includes local radiotherapy, anti-CD20 immunotherapy (e.g., rituximab), and combination chemoimmunotherapy. Active surveillance is used in selected patients with asymptomatic, low-burden disease. For patients with progressive disease, chemoimmunotherapy regimens such as obinutuzumab are standard [2]. While conventional therapies are often effective, their toxicity drives interest in safer, disease-modifying alternatives.

Emerging evidence has drawn attention to metabolic vulnerabilities that such strategies aim to exploit: cancer cells operate under chronically elevated oxidative stress relative to normal tissue as a consequence of altered metabolism and rapid proliferation, rendering them more susceptible to further disruption of antioxidant defenses, including altered amino acid dependencies and heightened sensitivity to oxidative stress relative to normal tissues [3,4]. Restriction of the sulfur-containing amino acids methionine and cysteine limits the availability of glutathione (GSH) precursors and has been shown in preclinical models to sensitize tumor cells to ferroptosis, a regulated form of iron-dependent cell death [5,6]. Therapeutic-dose sodium selenite has been proposed to compound this vulnerability by disabling the thioredoxin antioxidant system, and has been shown to induce apoptosis in lymphoma cells [7,8]. These approaches remain investigational and are not part of standard management.

We report a case of SS-associated bilateral parotid MALT lymphoma that progressed substantially over 28 months of observation and subsequently demonstrated rapid and sustained regression after initiation of such a protocol, without chemoimmunotherapy. To our knowledge, the clinical impact of a combined dietary, nutraceutical, and redox-modulating protocol in MALT lymphoma has not been previously described in the peer-reviewed literature.

## Case presentation

A 52-year-old woman presented with a seven-month history of progressive left parotid swelling. Review of systems was notable for chronic dry eyes and dry mouth (sicca symptoms), for which she was subsequently diagnosed with SS, confirmed on minor salivary gland (lip) biopsy. The patient had a positive history of autoimmune hemolytic anemia (AIHA), responsive to corticosteroids but unresponsive to two prior courses of rituximab, administered approximately 2.8 years and 1.5 years before the presentation, respectively. She had a positive family history for chronic lymphocytic leukemia (CLL). Taken together, progressive parotid swelling in a patient with sicca symptoms and an autoimmune background represented a recognizable presenting pattern for SS-associated MALT lymphoma; this prompted directed evaluation, including parotid gland imaging and biopsy, rather than attribution to sicca-related gland enlargement alone.

Physical examination at diagnosis, approximately 28 months before protocol initiation, revealed a firm 2 x 3 cm left parotid mass. Neck ultrasonography demonstrated a 1.6 x 2.9 cm hypoechoic left parotid lesion. Fluorodeoxyglucose (FDG) positron emission tomography-computed tomography (PET-CT) demonstrated pathologic FDG uptake in the left parotid mass, suspicious for lymphoma, with diffuse mild bilateral salivary gland uptake consistent with SS-associated sicca syndrome (Figure 1). It also showed indeterminate mildly elevated focal FDG uptake in the right parotid gland without a corresponding CT abnormality. Incisional biopsy of the left parotid lesion revealed histopathology and immunophenotyping consistent with low-grade B-cell marginal zone MALT lymphoma. Testing for *Helicobacter pylori* was negative.

**Figure 1:**
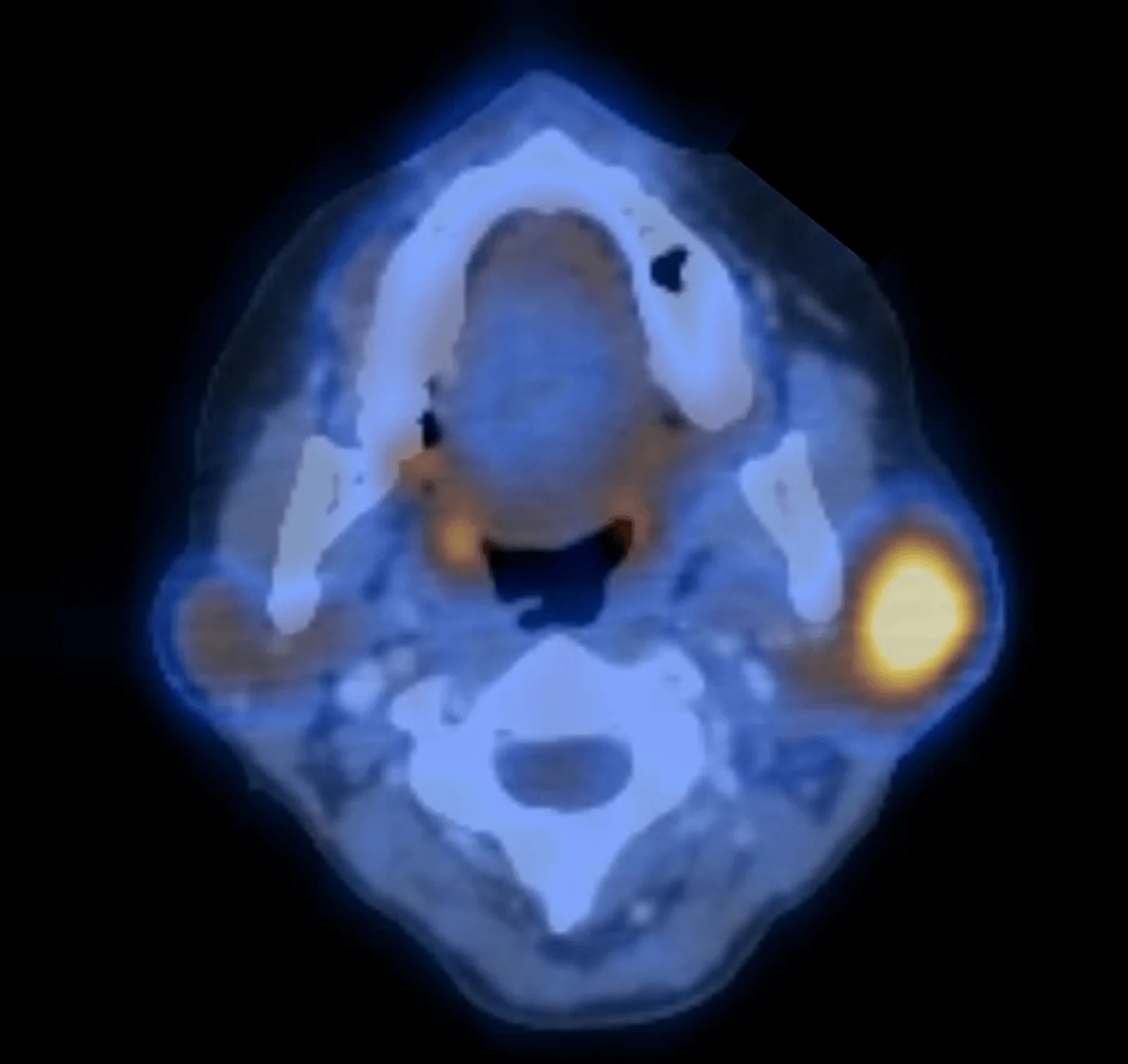
Axial fused FDG PET-CT image at the level of the parotid glands, obtained at diagnosis. Image demonstrates pathologic FDG uptake in the left parotid gland (image right, per radiological convention), corresponding to the biopsy-confirmed marginal zone lymphoma. Milder, indeterminate uptake is also present in the right parotid gland without a corresponding CT abnormality. FDG: fluorodeoxyglucose

The disease was staged as Lugano Stage II on the basis of clinically and radiographically apparent multi-site extranodal involvement (bilateral parotid and lacrimal gland enlargement). This staging is presumptive: only the left parotid lesion was histopathologically confirmed; the right parotid was not biopsied but showed diffusion-restricted lesions suspicious for lymphoma on MRI, and the lacrimal glands were enlarged on the same study but not biopsied, with imaging favoring a reactive rather than lymphomatous process. The biopsy-confirmed left parotid lesion, progressive bilateral parotid enlargement, and clinically evident lacrimal gland enlargement were documented by the treating hematologist as suspicious for lymphoma. Bone marrow biopsy was not performed; marrow involvement was therefore not assessed.

Given the indolent nature of the disease and the patient's preference, a strategy of active surveillance was adopted. The patient continued prednisone for management of her underlying AIHA. Over the next 28 months, physical examinations documented progressive bilateral parotid enlargement: the left parotid enlarged to a maximum palpable dimension of 10 x 5 cm and the right parotid reached 6 x 6 cm. Clinical photographs illustrate the degree of bilateral parotid enlargement at this stage (Figure 2A-2C). 

**Figure 2:**
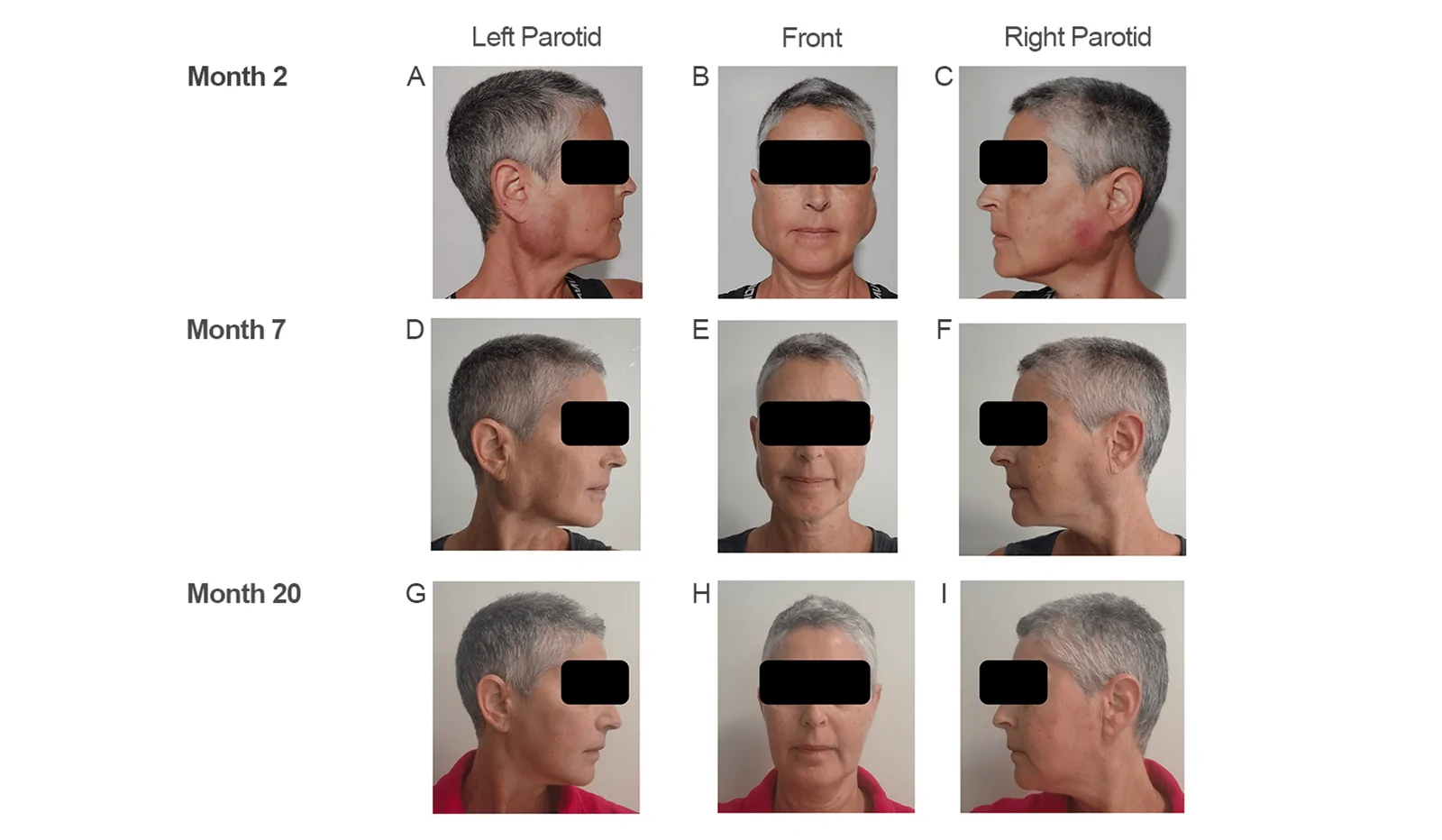
Sequential clinical photographs demonstrating bilateral parotid enlargement and regression (months measured from protocol initiation, Month 0). A–C (approximately Month 2): left parotid, frontal, and right parotid views, respectively, showing bilateral parotid enlargement near maximum extent, in the early weeks of protocol initiation and prior to any clinically evident response. Prominent left parotid mass visible on lateral view (A); erythema and swelling of right parotid region visible on contralateral view (C). D–F (approximately Month 7): corresponding views demonstrating marked reduction in bilateral parotid volume with restoration of facial contour. G–I (approximately Month 20): corresponding views demonstrating near-complete resolution of bilateral parotid swelling. Written consent to publish photographs obtained.

The treating team recommended chemoimmunotherapy, which the patient elected to defer. Approximately 28 months after initial diagnosis, the patient independently started a structured nutritional and nutraceutical protocol (Month 0) developed by the Nutritional Oncology Research Institute (NORI), the components of which are summarized in Table 1.

**Table 1:** Summary of nutritional and nutraceutical protocol components, dosing schedule, and proposed mechanisms. *Drawn from preclinical and early clinical literature and presented as a hypothesis-generating framework rather than established mechanisms of action in this patient. PUFA: polyunsaturated fatty acid; ROS: reactive oxygen species; GSH: glutathione; NK cell: natural killer cell; TXNRD1: thioredoxin reductase-1; P5P: pyridoxal-5′-phosphate; MnSOD: mitochondrial superoxide dismutase

Intervention	Daily dose/schedule	Proposed mechanism*
Dietary protocol: strict vegan diet, restricting methionine and cysteine; high fruit intake; low protein (~30-40 g/day isocaloric); 4 tsp PUFA oil (flaxseed, hemp) daily; no antioxidant supplements	Weekdays (5 days/week); methionine + cysteine target ≤4 mg/kg/day	Restricts cysteine and methionine as precursors for GSH synthesis; high-PUFA fatty acid profile proposed to destabilize cancer cell membranes and support ferroptosis; antioxidant supplements eliminated to avoid counteracting prooxidant effects [3-5,9]
Alkalization: potassium bicarbonate + sodium bicarbonate + magnesium carbonate	Titrated to maintain urinary pH ≥7.5 on daily self-monitoring; weekdays	Proposed modulation of tumor microenvironment pH; oral bicarbonate shown in preclinical models to raise tumor pHe and reduce metastatic spread [10], and to reactivate NK cell antitumor immunity in B-cell lymphoma [11]
Sodium selenite (SSe), slow-releasing, therapeutic dose	32 mg/day (8 mg × 4 doses, 5 days/week); taken with food	Inhibits TXNRD1, disabling the thioredoxin antioxidant system; induces apoptosis in lymphoma cells at therapeutic doses; 32 mg/day oral is within the range studied in published phase I clinical trials [7,8,12,13]
P5P; active vitamin B6	600 mg/day (150 mg × 4 doses, 5 days/week)	Enzyme-independent catabolism of residual cysteine via thiazolidine intermediate; further reduces substrate availability for GSH synthesis [14]
Iron (ferric ammonium citrate, FAC)	40 mg/day (10 mg × 4 doses, 5 days/week)	Fenton-reaction substrate; generates hydroxyl radicals; catalyst for P5P-cysteine reaction; potentiates ferroptotic cell death [5,14,15]
Copper gluconate	40 mg/day (10 mg × 4 doses, 5 days/week)	Redox-active transition metal; generates ROS via Fenton-like reactions; modulates antioxidant metalloenzyme activity at supraphysiological concentrations [16]
Zinc picolinate	60 mg/day (15 mg × 4 doses, 5 days/week)	Modulates antioxidant metalloenzyme activity and intracellular ROS signaling at supraphysiological concentrations [16]
Manganese	24 mg/day (6 mg × 4 doses, 5 days/week)	Cofactor for MnSOD; generates ROS via Fenton-like reactions; supraphysiological concentrations proposed to modulate antioxidant metalloenzyme activity [16]
Rectal ozone therapy	Starting at approximately month 3; 200 mL at 50 gamma (50 μg/mL), twice daily, five days per week; same days as nutraceutical protocol	Included for proposed prooxidant and pro-apoptotic activity within the protocol's redox-targeting framework; mechanistic data are preclinical and derived from non-lymphoma cell lines; clinical investigation in B-cell lymphoma warranted [17]
Weekend diet (2 days/week)	More varied vegan diet; increased protein (legumes, grains, pseudograins); continue PUFA oil; no nutraceuticals on weekend days; methionine + cysteine target ~10 mg/kg/day	Higher protein intake on weekend days is intended to support nutritional adequacy on a 5/2 cycling schedule.

The diet consisted of a strict vegan, isocaloric, low-protein diet (approximately 30-40 grams of protein per day) emphasizing fruits and vegetables. It specifically restricted the sulfur-containing amino acids methionine and cysteine (high in animal-based foods), with a weekday target of 4 mg/kg/day for each amino acid. Four teaspoons of high polyunsaturated fatty acid (PUFA) oil (flaxseed and hemp) were consumed daily, with avoidance of monounsaturated fat sources such as avocado and olive oil, to support ferroptotic membrane destabilization [5,9]. All antioxidant supplements were eliminated. The patient monitored urinary pH daily as an indicator of tumor microenvironment pH. Orally administered nutraceuticals, initiated shortly after the dietary protocol, included slow-releasing sodium selenite at a therapeutic dose of 32 mg/day, pyridoxal-5'-phosphate (P5P), which is the active form of vitamin B6, at 600 mg/day, iron (ferric ammonium citrate) at 40 mg/day, copper gluconate at 40 mg/day, zinc picolinate at 60 mg/day, and manganese at 24 mg/day (see Table 1 for dose and schedule). All were taken in four divided doses five days per week. On weekends, nutraceuticals were withheld, and dietary protein intake was increased to maintain nutritional adequacy. At approximately Month 3, the patient additionally began rectal ozone therapy at a dose of 200 mL at 50 gamma (50 μg/mL), twice daily, five days per week. Prednisone dosing fluctuated throughout the observation for management of the patient's AIHA, ranging from 0 to 30 mg daily, with a daily average of 8 mg.

At approximately Month 7, neck ultrasonography showed a right parotid hypoechoic lesion measuring 2.7 cm and a left parotid hypoechoic heterogeneous lesion measuring 4.9 × 1.5 cm (Figure 3A, 3B); clinical photographs taken the same day document the corresponding bilateral parotid volume (Figure 2D-2F). The treating hematologist documented progressive improvement in parotid and lacrimal gland size on serial clinical examinations from Month 8 onward. At approximately Month 19, the treating hematologist documented no significant lymphadenopathy or splenomegaly on clinical examination, consistent with the ongoing improvement noted above. At approximately Month 20, repeat neck ultrasonography confirmed substantial radiographic reduction: the right parotid lesion decreased to 1.5 × 0.8 cm and the left to 2.7 × 0.8 cm (Figure 3C, 3D), with both glands returning to normal overall size, residual hypoechoic lesions, and no cervical lymphadenopathy. Clinical photographs taken at the same time demonstrate near-complete resolution of bilateral parotid swelling with restoration of normal facial contour (Figure 2G-2I). Based on available data, the response was categorized as a partial response by imaging-limited Lugano criteria, which do not incorporate the metabolic (FDG PET-CT) or bone marrow assessment required to confirm a complete response; formal assessment by FDG PET-CT was declined by the patient. At approximately Month 24, the patient continues to adhere to a modified protocol with sustained clinical improvement.

**Figure 3:**
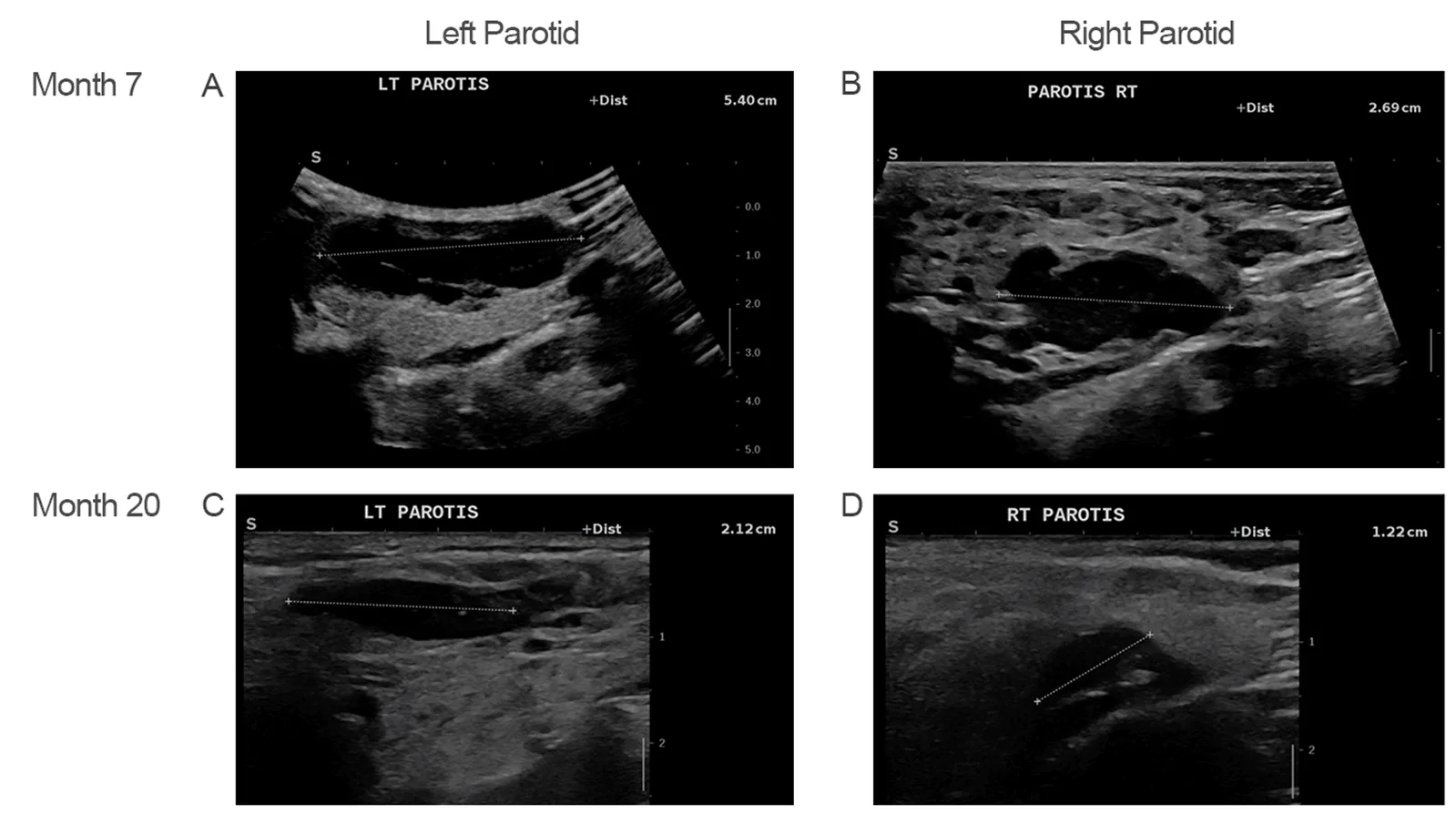
Sequential grayscale ultrasound images of the left and right parotid glands demonstrating interval reduction in lesion size. A and B (approximately Month 7): Hypoechoic lesions measuring 5.4 cm (left) and 2.7 cm (right). C and D (approximately Month 20): Interval reduction to 2.1 cm (left) and 1.2 cm (right). Caliper (+Dist) measurements are annotated on each image. Caliper measurements reflect a single plane (most consistent with a transverse view) captured per still image and may differ modestly from the two-dimensional length-and-width measurements reported in the corresponding radiology report.

Throughout the observation period, the patient maintained stable body weight and normal albumin, providing indirect evidence of preserved nutritional status. Serial laboratory investigations during the protocol period demonstrated that liver function, kidney function, and electrolytes remained within normal limits throughout, with no hepatotoxicity signal detected. White blood cell count and platelet count were in range.

This protocol was developed and administered with guidance from the NORI, outside the supervision of the patient's treating hematology-oncology team and outside a formal clinical trial or research protocol. It is reported here as a clinical observation, not as a treatment recommendation. Several components were used at doses exceeding established safety thresholds and carrying risk of toxicity (see Limitations).

## Discussion

MALT lymphoma arising in the parotid gland in the setting of SS is an indolent entity for which active surveillance is appropriate in stable, low-burden disease [2]; however, progressive bilateral enlargement over 28 months, as in this case, warrants systemic treatment [2]. After the treating team recommended chemoimmunotherapy, the patient elected to defer and initiated a multicomponent nutritional and nutraceutical protocol. The subsequent rapid and sustained regression of bilateral parotid masses over two years represents a notable and unusual clinical course.

Biological rationale for the proposed protocol

The mechanisms proposed below draw on a growing preclinical literature on redox vulnerabilities in cancer cells and are offered as a hypothesis-generating framework for the observed clinical course, rather than as established mechanisms in this patient.

The protocol was designed to selectively increase reactive oxygen species (ROS) within malignant cells by simultaneously targeting two major intracellular antioxidant defense systems: the GSH pathway and the thioredoxin pathway. Cancer cells operate under chronically elevated oxidative stress relative to normal tissues as a consequence of altered metabolism, mitochondrial dysfunction, and rapid proliferation [3,4]. This elevated oxidative stress renders cancer cells particularly vulnerable to additional oxidative insults that overwhelm residual antioxidant capacity, a proposed selective therapeutic target [3,4].

The dietary component restricted methionine and cysteine through a strict plant-based regimen. These sulfur-containing amino acids serve as rate-limiting precursors for GSH synthesis; their dietary restriction impairs the primary intracellular buffer against ROS and has been shown in preclinical models to sensitize tumor cells to ferroptosis [5,6]. The high-PUFA fatty acid profile achieved through flaxseed and hemp oil is proposed to further promote ferroptotic membrane vulnerability. Polyunsaturated fatty acids incorporated into membrane phospholipids are the primary substrates for lipid peroxidation and have been shown to sensitize cancer cells to ferroptosis [5,9].

Sodium selenite has been shown to inhibit thioredoxin reductase-1 in vitro, converting the enzyme into a pro-oxidant that generates ROS rather than neutralizing them, disabling a major compensatory redox pathway in cancer cells [8,12]. In a randomized study (n=40) of newly diagnosed NHL patients, adjuvant sodium selenite significantly increased the percentage of apoptotic lymphoma cells compared with chemotherapy alone (78.9% versus 58.9%; p<0.05), providing preliminary clinical evidence of sodium selenite-induced lymphoma cell apoptosis in vivo; however, the randomization method and allocation concealment procedure are not described [7].

P5P, the active form of vitamin B6, has been shown to react with cysteine in an enzyme-independent manner, further reducing substrate availability for GSH synthesis [14]. Iron has been shown to catalyze this same non-enzymatic reaction, providing a proposed mechanistic rationale for combining both agents in this protocol [15]. Iron supplementation may additionally potentiate ferroptotic signaling through Fenton-type radical chemistry [5]. Copper, zinc, and manganese were included for their roles as redox-active metals capable of generating ROS via Fenton-like reactions and modulating antioxidant metalloenzyme activity at supraphysiological concentrations [16].

The alkalization component, titrated to maintain urinary pH at or above 7.5, was intended to modify the characteristically acidic tumor microenvironment. Oral bicarbonate administration has been shown in preclinical models to selectively raise tumor extracellular pH and reduce metastatic spread [10] and to reactivate NK cell-mediated antitumor immunity specifically in a B-cell lymphoma model [11]; clinical evidence for oral bicarbonate alkalization in MALT lymphoma is currently absent.

At approximately Month 3, the patient added rectal ozone therapy (see Table 1 for dose and schedule). Ozone therapy was included for its proposed capacity to introduce controlled oxidative stress and potentiate mitochondrial-mediated apoptosis, providing an additional prooxidant challenge within the protocol's redox-targeting framework [17]; mechanistic data are currently preclinical, and its specific role in B-cell lymphoma warrants further investigation.

Taken together, the components of this protocol are hypothesized to act synergistically: dietary restriction to reduce GSH precursor availability, P5P to catabolize residual cysteine, sodium selenite to disable the thioredoxin compensatory pathway, alkalization to modify the tumor microenvironment, and ozone therapy to impose additional prooxidant challenge. Collectively, these components are hypothesized to drive ROS to levels sufficient to trigger apoptotic and ferroptotic tumor cell death.

Nutraceutical dose and safety

The slow-releasing sodium selenite dose employed in this case, 32 mg/day orally administered in four equal doses, represents a therapeutic rather than supplemental dose. Sodium selenite has an elemental selenium content of approximately 45.7% by molecular weight; 32 mg/day therefore corresponds to approximately 14,600 μg elemental selenium on dosing days. While this substantially exceeds the Institute of Medicine tolerable upper intake level of 400 μg/day established for chronic dietary supplementation [18], it is consistent with doses evaluated in published phase I clinical trials of oral sodium selenite in cancer patients [13]. In a phase I study of sodium selenite combined with palliative radiotherapy, no dose-limiting toxicities were observed at any dose up to 49.5 mg/day, with only grade 1 gastrointestinal side effects at the 33 mg/day level. Investigators concluded that doses up to 33 mg/day were well tolerated [13]. For context, acute selenium toxicity has been documented at exposures of approximately 41,700 μg/day, approximately 2.9-fold higher than the elemental content of the dose in this case, in instances of supplement misformulation [19].

The patient reported no clinical signs of selenosis throughout the two-year observation period, including no hair loss, nail changes, or neurological symptoms. Serial laboratory monitoring throughout the protocol period demonstrated preserved liver and kidney function at all time points, with no hepatotoxicity signal detected despite therapeutic-dose sodium selenite administration over 18 months. Albumin remained normal throughout, and estimated glomerular filtration rate (eGFR) was stable at 94-101 mL/min/1.73m². Electrolytes, including potassium, were within normal limits, supporting the safety and nutritional adequacy of the protocol as implemented in this patient. The administered dose should not be interpreted as a recommendation; administration of sodium selenite at therapeutic doses requires appropriate clinical supervision.

P5P was administered at 600 mg/day, sixfold the Institute of Medicine tolerable upper intake level of 100 mg/day for vitamin B6 in adults [18]; sensory peripheral neuropathy is the primary dose-dependent risk associated with prolonged high-dose vitamin B6 exposure. The patient reported no neurological symptoms throughout the two-year protocol period, and no formal neurological assessment was performed. Copper gluconate, zinc picolinate, and manganese were administered at doses exceeding the Institute of Medicine tolerable upper intake levels [18] by approximately four-fold, 1.5-fold, and two-fold, respectively. Known risks include hepatotoxicity and hemolytic anemia (copper), copper depletion via metallothionein induction (zinc), and neurological toxicity (manganese). Serum copper was within the normal reference range at six weeks (86.5 mcg/dL; reference 65-165 mcg/dL); serum zinc and manganese were not measured. Liver function remained within normal limits at all assessment timepoints, and the patient reported no symptoms attributable to toxicity of any of these agents throughout the two-year observation period.

Spontaneous regression

Spontaneous regression of indolent B-cell lymphomas must be considered in interpreting this case [20,21]. In the landmark natural history study of 83 untreated patients with low-grade NHL under observation, spontaneous regression occurred in 23%, typically as partial and transient waxing-and-waning events rather than sustained unidirectional responses [21]. In the largest published series of salivary gland MALT lymphoma, a multicenter international study of 247 patients (International External Lymphoma Study Group Trial 41), the clinical course was characterized by an overall indolent trajectory, with no spontaneous regression events specifically documented during progressive disease [20]. Bilateral involvement, as seen in this patient, was associated with more advanced disease requiring systemic consideration.

Several features argue against spontaneous regression: (i) 28-month documented continuous bilateral progression prior to protocol initiation, reaching maximum dimensions of 10 x 5 cm on the left and 6 x 6 cm on the right; (ii) rapid subsequent regression, unidirectional, and sustained over two years without fluctuation; and (iii) a directional change that coincides temporally with protocol initiation (Figure 4), rather than the waxing-and-waning pattern characteristic of spontaneous regression in indolent lymphoma [21]. No clinical features suggestive of histologic transformation were observed during the follow-up period.

**Figure 4:**
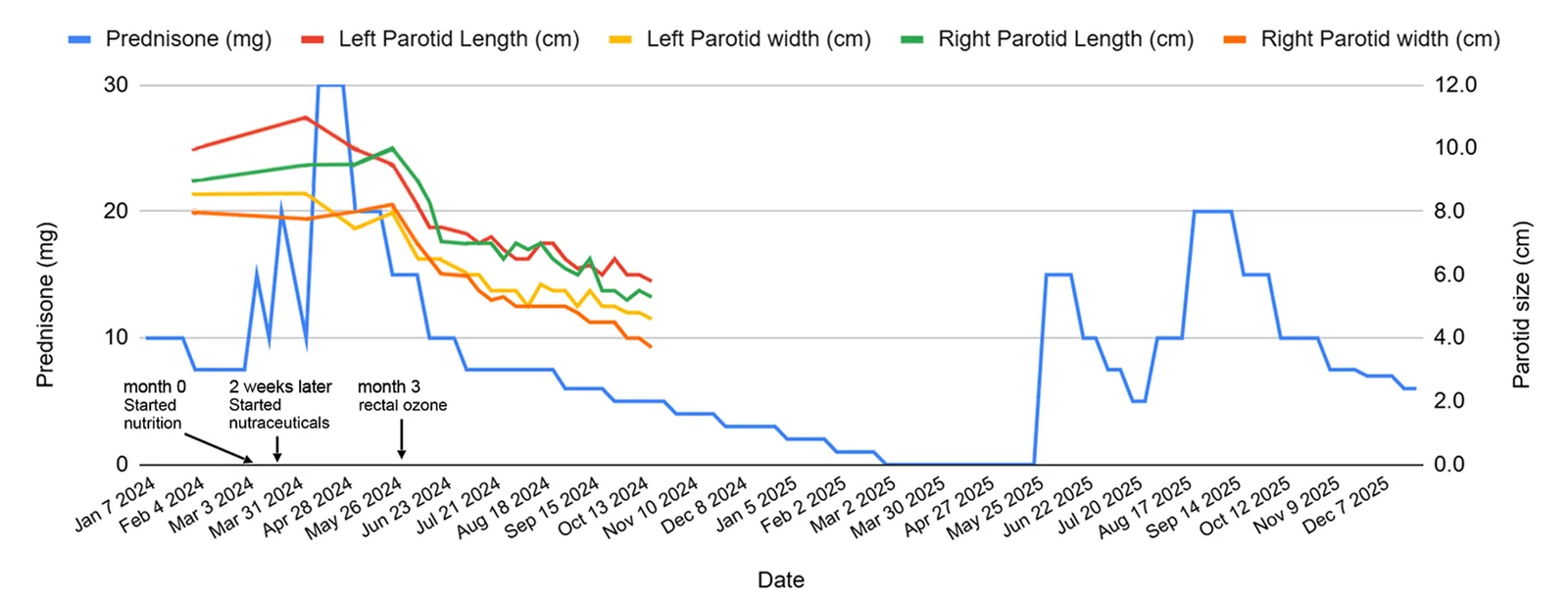
Parotid dimensions over time compared with prednisone dosage Dual-axis plot showing left parotid length (red), left parotid width (yellow), right parotid length (green), and right parotid width (orange) measured by the patient over time using a flexible tape measure, plotted against concurrent prednisone dose in milligrams (blue, left axis); parotid measurements in centimeters on the right axis. Annotated arrows indicate protocol milestones: dietary protocol initiation (Month 0), nutraceutical supplementation (approximately two weeks later), and rectal ozone therapy (Month 3). Progressive parotid regression is apparent following protocol initiation and is sustained through the most recent follow-up (approximately Month 21), independent of prednisone dose fluctuations. Patient-performed measurements using a flexible tape measure carry inherent variability and should be interpreted accordingly; independent clinical palpation findings by the treating hematologist at key time points are reported in the text and are consistent with the measurement trend shown.

Role of prednisone

The patient received prednisone for AIHA both before and throughout the protocol period (0-30 mg daily). Corticosteroids exert lympholytic effects and could in principle contribute to lymphoid tissue reduction. Notably, prednisone was maintained throughout the preceding 28-month surveillance period, during which the glands enlarged progressively despite steroid exposure, arguing against prednisone as sufficient explanation for the later regression. Patient-performed measurements (Figure 4) suggest regression beginning around Month 3, before formal clinical confirmation at Month 7-8; prednisone was tapering but still active during this interval, so an early corticosteroid contribution cannot be excluded. Prednisone was stopped entirely from approximately Month 12 to Month 15 (~12 weeks); the hematologist documented improvement within this off-steroid window, the clearest evidence against a corticosteroid-mediated cause. Prednisone was subsequently resumed (15-20 mg) through approximately Month 18, with regression continuing to near-complete resolution by Month 20 (Figure 2G-2I), a period that does not itself distinguish between mechanisms. Taken together, 28 months of progression under steroid exposure and 12 weeks of regression during steroid cessation provide the strongest evidence against a corticosteroid-mediated cause, though a partial early contribution cannot be excluded.

Limitations

Several limitations should be noted. This case should be interpreted with the understanding that histopathologic confirmation was obtained from the left parotid lesion only. Biopsy of the right parotid mass was not considered during the diagnostic workup, but it showed diffusion-restricted lesions suspicious for lymphoma on MRI. Although SS-associated lymphoepithelial sialadenitis (LESA) cannot be formally excluded for the right-sided lesion, the 28-month progressive bilateral enlargement, focal FDG uptake in the right parotid on PET-CT, and parallel bilateral regression tracking precisely with protocol initiation are more consistent with bilateral lymphomatous involvement.

Response assessment was based on clinical examination, patient-performed measurements, and ultrasonography rather than repeat FDG PET-CT; the patient declined further nuclear imaging, and no additional imaging is planned. Patient-performed tumor measurements using a flexible tape measure carry inherent variability, though these are supported by independent hematologist examination findings at multiple time points. Where measurements diverged across sources, radiology report values were treated as the primary evidence, with clinical examination, patient-performed measurements, and photographic documentation regarded as corroborative rather than primary.

The multicomponent nature of the intervention, with components introduced sequentially, means that the contribution of any individual element cannot be determined.

## Conclusions

This report describes the case of a 52-year-old woman who presented with progressive bilateral parotid extranodal marginal zone MALT lymphoma and who achieved durable, near-complete clinical and ultrasound-based regression without conventional chemoimmunotherapy. The intervention consisted of a strict vegan diet restricting methionine and cysteine, urinary alkalization, and orally administered nutraceuticals at therapeutic doses, including sodium selenite, P5P, iron, copper, zinc, and manganese, with adjunctive rectal ozone therapy. The observed regression is hypothesized to reflect tumor cell death mediated through apoptotic and ferroptotic pathways driven by simultaneous disruption of GSH and thioredoxin antioxidant defense systems.

The rapidity and durability of this regression in the setting of previously progressive bilateral disease, sustained over two years without conventional systemic therapy, is markedly unusual, though spontaneous regression and fluctuating prednisone exposure cannot be entirely excluded as contributing factors. These findings support further investigation into the potential role of dietary and redox-modulating strategies in patients with indolent B-cell lymphomas. However, as this report describes a single patient's clinical course, supported by guidance from a nutritional oncology organization outside the patient's treating oncology team, it should not be interpreted as evidence of efficacy or as a treatment recommendation. The doses of selenium, vitamin B6, and trace minerals used in this protocol substantially exceed established safety thresholds, and self-administration without medical supervision carries a serious risk of toxicity.
